# Antioxidant Potential, DNA Protection, and HPLC-DAD Analysis of Neglected Medicinal *Jurinea dolomiaea* Roots

**DOI:** 10.1155/2014/726241

**Published:** 2014-05-28

**Authors:** Naseer Ali Shah, Muhammad Rashid Khan, Kiran Naz, Mubarak Ali Khan

**Affiliations:** ^1^Department of Biochemistry, Faculty of Biological Sciences, Quaid-i-Azam University, Islamabad 45320, Pakistan; ^2^Department of Biotechnology, Faculty of Biological Sciences, Quaid-i-Azam University, Islamabad 45320, Pakistan

## Abstract

*Jurinea dolomiaea* Boiss., family Compositae, is a medicinally important plant of alpine region. Its tuberous roots are used in various ailments in folk medicine. This study was undertaken to estimate total phenolic (TPC) and total flavonoid contents (TFC) and to determine anti-free radical potential by diverse *in vitro* antioxidant assays. Crude methanol extract (JDME) was fractionated into *n*-hexane (JDHE), chloroform (JDCE), ethyl acetate (JDEE), *n*-butanol (JDBE), and aqueous (JDAE) fractions. The results indicated that JDEE and JDCE constituted the highest amount of TFC (807 ± 7.2 mg rutin equivalent/g sample) and TPC (757 ± 9.4 mg gallic acid equivalent/g sample), respectively. Significant correlation of TFC with IC_50_ values was recorded for ^•^OH (*R* ^2^ = 0.91), H_2_O_2_ (*R* ^2^ = 0.82), and ABTS (*R* ^2^ = 0.82) assay. It could be made clear that JDEE was the most potent in antioxidant activity as compared to others, with generally lower IC_50_ values for DPPH (41.1 ± 1.0 **μ**g/mL), ABTS (46.7 ± 0.6 **μ**g/mL), H_2_O_2_ (42.2 ± 0.9 **μ**g/mL), ^•^OH (61.1 ± 1.1 **μ**g/mL), O_2_^−^ (152 ± 1.1 **μ**g/mL), and antilipid peroxidation (54.3 ± 1.6 **μ**g/mL). HPLC chromatogram of JDEE revealed the presence of catechin, caffeic acid, and rutin. The results indicated the antioxidant activities of *J. dolomiaea* roots and merit further investigations for their use in oxidative stress related disorders.

## 1. Introduction

Free radicals such as reactive oxygen species (ROS) and reactive nitrogen species (RNS) are classified having (or not) one or more unpaired electron. They belong to very reactive species, produced continuously in cells as normal metabolic products. They have both beneficial as well as harmful roles in living body. ROS play an important role in physiological mechanisms such as induction of mitogenic responses, cellular signaling pathways, and responses to infectious agents at low and moderate concentrations, while RNS play an important role in blood pressure maintenance, neurotransmission, defense mechanism, immune regulation, and smooth muscle relaxation. In biological systems, the overproduction of ROS and RNS results in state termed oxidative stress and nitrosative stress, respectively. However, protective efficiency depends upon the balance between ROS/RNS and enzymatic or nonenzymatic antioxidants in the microenvironment of the cell. So an imbalance induces deteriorative actions like damage of cellular proteins, lipids, and DNA. If this state continues for a longer period, it may induce or enhance many clinical disorders such as inflammatory diseases, aging, asthma, diabetes mellitus, cardiovascular diseases, rheumatoid arthritis, and cancer [1] and neurodegenerative disorders such as Parkinson's and Alzheimer's diseases [2].

In addition to health hazards, oxidation is also playing a deleterious role in the food industry which may lead to the degradation of lipids and proteins and contribute to the deterioration in flavor, texture, and color of food products [3–5]. Nowadays, the market is full of neutralizing agents of free radicals called antioxidants. But most of them are synthetically manufactured such as butylated hydroxytoluene (BHT) or butylated hydroxyanisole (BHA) in food industry [3]. But their value has been questioned due to associated problems like toxicity and/or mutation induction [6]. Natural antioxidant, including ascorbic acid and tocopherol, is less effective than synthetic antioxidants. Hence there is a need to identify new natural antioxidants for use as safe and effective additive in the food industry [7].

For natural antioxidants, plants have gained much attention for producing compounds with antioxidant property, showing protection against oxidative stress. In plants, secondary metabolites such as flavonoids are a well known class of antioxidants [8], due to their high redox value, making them a good hydrogen donor, reducing agent, and singlet oxygen scavenger [9]. In addition to health benefits, flavonoids are well recognized for their character to hinder oxidative damage of unsaturated fatty acids; rapid and simple metabolic degradation pathways make them an ideal preservative alternative to synthetic antioxidants in food industry [3].

Antioxidant activity is a complex process usually occurring through several mechanisms. Taking into consideration the difference among the broad number of antioxidant assays, the results of a single assay can give only a limited implication of the antioxidant capacity of extracts towards food item and must be interpreted with some care. Extract/fractions are often a mixture of complex chemical entity, having a dozen of compounds with different chemical nature and polarity, leading to diverse results depending on assay systems in use. Therefore, an approach of multiple assays in plant antioxidant potential screening work is highly desirable [10].

According to the chemical reaction used, methods to measure antioxidant capacity can be mainly grouped into 2 classes: hydrogen atom transfer (HAT) and electron transfer (ET) based methods [11]. HAT methods measure the ability of an antioxidant to quench free radicals by hydrogen donation. In most HAT methods, antioxidants and a probe compete for thermally generated peroxyl radicals and the quantitation is derived from the kinetic curves after monitoring the competitive reaction kinetics. ET methods measure the ability of a potential antioxidant to transfer 1 electron to reduce radicals, metals, or carbonyls, which use the color change of the oxidant as the endpoint indicator. Because multiple reaction mechanisms and different phase locations are usually involved in measuring the antioxidant capacity of a complex extract/fraction system, there is no simple universal method by which “total antioxidant capacity” can be measured accurately and quantitatively [11–16].

Therefore, multiple antioxidant assays with different mechanisms, such as scavenging of DPPH radical, superoxide anion (O_2_^•^) radical, hydroxyl radical (^•^OH) radical, hydrogen peroxide (H_2_O_2_), nitric oxide (NO^•^) radical, ABTS radical, *β*-carotene, and reducing power assay, phosphomolybdenum assay, and antilipid peroxidation assay were performed. DNA protection assay was also evaluated to explore extract and different fraction antioxidant activities against hydroxyl radical generated by Fenton reaction.

*Jurinea dolomiaea* synonym* Jurinea macrocephala,* family Compositae, is an economically important plant having long tuberous roots. It flowers from July to October. Its habitat is in rock crevices, exposed slopes, and glacial moraines between 3000 and 4500 meters in alpine region (Kohitan, Pakistan) and also reported from Byas and Darma valley. The roots of the plant are used for making binding substance known as gum. Roots are used as antiseptic and applied as poultice on eruptions and decoction is given in colic. It is also considered cordial and is given in puerperal fevers. Roots are considered to be stimulant and given in fever after child birth [17, 18]. Aromatic oil from the roots is useful in eye infection, rheumatism gout [19] incense, diarrhea, and stomachache [20].

At present, there is no single study regarding its phytochemical constituents and antioxidant potential. So this study was undertaken to evaluate its chemical constituents and antioxidant activities.

## 2. Materials and Methods

### 2.1. Plant Collection and Preparation of Extract

*Jurinea dolomiaea* roots were collected in the month of September 2012 from District Kohistan, Pakistan, and were recognized by their local names and then confirmed by Professor Dr. Mir Ajab Khan, Department of Plant Sciences, Quaid-i-Azam University, Islamabad, and Dr. Muhammad Zafar, Curator, Herbarium, Quaid-i-Azam University, Islamabad. Voucher specimen with Accession number 27823 was deposited at the Herbarium, Quaid-i-Azam University, Islamabad.

Shade dried 5 Kg powder of* J. dolomiaea roots* was extracted twice for 72 h in 10 liters of crude methanol and filtered through filter paper (Whatman filter paper number 45), and the filtrate was concentrated through rotary vacuum evaporator at reduced pressure to get crude methanol extract (JDME). To sort the extract in increasing order of polarity it was suspended in distilled water (6 g/250 mL) and passed through different solvents (250 mL each) in the order of* n*-hexane (JDHE) →chloroform (JDCE)→ethyl acetate (JDEE)→*n*-butanol (JDBE) to get different fractions by using separating funnel. The soluble residue was termed aqueous fraction (JDAE). All the fractions were stored at 4°C until further use.

### 2.2. Phytochemical Analysis

#### 2.2.1. Total Phenolic Content (TPC) Estimation

Spectrophotometric method [21] was used with minor modifications to determine total phenolic content. In short, 200 *μ*L of the sample (1 mg/mL) was mixed with 1 mL of the 1 : 10 Folin-Ciocalteu's reagent. To the mixture 2.5 mL of 7% Na_2_CO_3_ was added after 5 min. The mixture was incubated at 23°C in the dark for 90 min. Absorbance was recorded at 765. Total phenolic content was calculated from calibration curve of gallic acid. Estimation of TPC was recorded in triplicate and presented as mg of gallic acid equivalents (GAE) per g of dry sample.

#### 2.2.2. Total Flavonoid Content (TFC) Estimation

In a test tube, 0.3 mL of sample, 3.4 mL of 30% methanol, 0.15 mL of 0.5 M NaNO_2_, and 0.1 mL of 0.3 M AlCl_3_ · 6H_2_O were thoroughly mixed. After 5 min, 1 mL of 1 M NaOH was added and mixed well. Absorbance was measured at 506 nm against the reagent blank. Total flavonoid content was estimated by using a calibration curve of rutin and expressed as mg rutin equivalents per g of dry sample [22].

### 2.3. HPLC Analysis

Methanol, acetonitrile, and acetic acid were of HPLC grade (Tedia Company, USA), while deionized water was prepared by a Milli-Q Water Purification system (Millipore, MA, USA). Nine reference standards were used, that is, catechin, rutin, kaempferol, quercetin, gallic acid, salicylic acid, apigenin, myricetin, and caffeic acid (Sigma company, USA). Standards and plant extract stock solutions were prepared in methanol, at a concentration of 200 *μ*g/mL and 10 mg/mL, respectively. Samples were filtered through 0.45 *μ*m membrane filter.

Chromatographic analysis was carried out by using HPLC-DAD attached with Agilent RP-C8 analytical column. Briefly, mobile phase A was acetonitrile-methanol-water-acetic acid (5 : 10 : 85 : 1) and mobile phase B was acetonitrile-methanol-acetic acid (40 : 60 : 1). A gradient of time 0–20 min for 0 to 50% B, 20–25 min for 50 to 100% B, and then isocratic 100% B till 40 minutes was used. The flow rate was 1 mL/min and injection volume was 20 *μ*L. All the samples were analyzed at 257, 279, 325, and 368 nm wavelength. Every time column was reconditioned for 10 min before the next analysis. All samples were assayed in triplicate. Quantification was carried out by the integration of the peak using the external standard method. All chromatographic operations were carried out at ambient temperature.

### 2.4. Antioxidant Capacity Determination Assays

An amount of 200 *μ*g of each sample and positive control (ascorbic acid, butylated hydroxyanisole, butylated hydroxytoluene, catechin, and gallic acid) was prepared in 1 mL of solvent according to the assay protocol. These solutions were further diluted to 100, 50, 25, and 12.5 *μ*g/mL. Positive control varied according to assay requirement.

#### 2.4.1. DPPH Radical Scavenging Assay

The DPPH (1,1-diphenyl-2-picrylhydrazyl) bioassay was performed according to the protocol of Ahmad et al. [23]. Working solution of DPPH with absorbance of 0.98 ± 0.02 was obtained at 517 nm from the standard solution of DPPH (0.24 mg/mL). A volume of 0.9 mL of DPPH solution was mixed well with 100 *μ*L of various concentrations of test samples and incubated for 30 min in the dark at room temperature. Absorbance was recorded at 517 nm. Scavenging activity was calculated using the following equation:

(1) $$\begin{array}{c} \text{Scavenging}\;\text{effect}\;\left(\%\right)=\left[\frac{\left(\text{control}-\text{sample}\right)}{\left(\text{control}\right)}\right]\times 100. \end{array}$$

Ascorbic acid was used as positive control.

#### 2.4.2. Hydrogen Peroxide Scavenging Assay

The method of Sahreen et al. [24] was followed to investigate hydrogen peroxide scavenging ability of samples. A solution of 40 mM hydrogen peroxide was prepared in 50 mM phosphate buffer (pH 7.4). Samples (0.4 mL) in distilled water were added to a hydrogen peroxide solution (0.6 mL). Absorbance of the mixture was recorded at 230 nm after 10 min of incubation later against a blank solution containing the phosphate buffer without hydrogen peroxide. Percent scavenging activity was calculated by following formula:

(2) %  scavenging  activity  =(1−absorbance  of  sampleabsorbance  of  control)×100.

Ascorbic acid served as positive control.

#### 2.4.3. Hydroxyl Radical Scavenging Assay

Hydroxyl radical scavenging activity was evaluated by the reported method of Zeng et al. [21]. Phosphate buffered saline (0.2 M, pH 7.4) was used as solvent in this assay. Sample solution (0.1 mL, 50–250 g/mL) was mixed with ferrous ammonium sulfate solution (0.2 mL, 0.4 mM), ascorbic acid solution (0.05 mL, 2.0 mM), H_2_O_2_ solution (0.05 mL, 20 mM), and mixed solution (0.6 mL, containing 2.67 mM of deoxyribose and 0.13 mM of EDTA) and incubated at 37°C for 15 min. Then, thiobarbituric acid solution (1 mL, 1%, w/v) and trichloroacetic acid solution (1 mL, 2%, w/v) were added. The mixture was boiled in water bath for 15 min and cooled in ice, and its absorbance was measured at 532 nm. The scavenging activity was calculated as

(3) Scavenging  effect  (%)  =[(control  absorbance−sample  absorbance)(control  absorbance)]   ×100.

BHA was used as the positive control.

#### 2.4.4. ABTS Radical Cation Scavenging Activity

Saeed et al. [25] methodology with slight modification was followed for ABTS (2,2 azobis, 3-ethylbenzothiazoline-6-sulphonic acid) radical scavenging activity. ABTS (7 mM) solution was reacted with 2.45 mM potassium persulfate in pure water and was kept overnight in dark for generation of dark colored ABTS radicals. For the assay, solution was diluted with pure water for an initial absorbance of 0.7 ± 0.005 at 745 nm. To determine the ABTS radical scavenging activity, 0.1 mL of each sample was mixed with 1 mL of ABTS solution in glass cuvette. Absorbance was measured after 6 min of mixing. Percent inhibition was calculated by following formula:

(4) Scavenging  effect(%)  =[(control  absorbance−sample  absorbance)(control  absorbance)]   ×100.

BHT was used as the positive control.

#### 2.4.5. Antilipid Peroxidation Assay

This assay was performed as illustrated by Dorman et al. [26]. A mixture of egg yolk (10%, w/v) was prepared in KCl (1.15%, w/v). It was homogenized for 30 sec and subsequently subjected to ultrasonication for 5 min. A volume of 0.1 mL of the samples was added in 0.5 mL of yolk homogenate and volume was made up to 1 mL with distilled water. 1.5 mL of 20% acetic acid (v/v; pH 3.5), ferric chloride (0.05 mL), and 1.5 mL of thiobarbituric acid (0.8%, w/v) dissolved in sodium dodecyl sulphate (1.1%, w/v) were thoroughly mixed and incubated for 60 min in a water bath.* n-*Butanol (5 mL) was added after cooling at room temperature, stirred, and then centrifuged for 15 min at 5000 rpm. The absorbance of supernatant was recorded at 532 nm. Catechin was used as positive control.

The percent antilipid peroxidation activity was determined by formula (1 − *S*/*C*) × 100, where *C* = absorbance of control and  *S* = absorbance of test sample.

#### 2.4.6. *β*-Carotene Bleaching Assay

Khan et al. [27] method was used for *β*-carotene bleaching assay. *β*-Carotene (2 mg) was dissolved in 10 mL of chloroform and mixed with 20 mg of linoleic acid and 200 mg of Tween 80 followed by chloroform removal under nitrogen. Subsequently, 50 mL of distilled water was mixed vigorously to prepare *β*-carotene linoleate emulsion. An aliquot of each sample (50 *μ*L) was mixed with 1 mL of the emulsion, thoroughly mixed, and absorbance was determined at 470 nm immediately against the blank solution. Capped tubes were then kept in a water bath at 45°C for 2 h and the difference with the initial reading was calculated by measuring the reading after 2 h. *β*-Carotene bleaching inhibition was estimated as the following equation:

(5) $$\begin{array}{c} \text{Bleaching}\;\text{inhibition}\left(\%\right)=\left[\frac{A_{0t}-A_{120t}}{A_{0c}-A_{120c}}\right]\times 100. \end{array}$$

BHT was used as the positive control.

#### 2.4.7. Superoxide Anion Radical Scavenging Assay

Riboflavin light NBT system assay was followed for superoxide radical scavenging activity [25]. The reaction mixture contained 0.5 mL of 50 mM phosphate buffer (pH 7.6), 0.3 mL of 50 mM riboflavin, 0.25 mL of 20 mM PMS, and 0.1 mL of 0.5 mM NBT, prior to the addition of 1 mL of each sample. Florescent lamp was used for starting the reaction. Absorbance was recorded at 560 nm after incubation of 20 min under light. The percent inhibition of superoxide anion generation was calculated using the following formula:

(6) Percent  scavenging  activity(%)  =(1−absorbance  of  sampleabsorbance  of  control)×100.

#### 2.4.8. Nitric Oxide Radical Scavenging Activity

Ebrahimzadeh et al. [28] protocol was used for estimation of nitric oxide scavenging activity. This protocol was based on the principle that sodium nitroprusside at physiological pH in an aqueous solution and aerobic condition generates nitric oxide which further reacts with oxygen to form nitrite ions, which is estimated by using Griess reagent. Scavengers of nitric oxide react with oxygen, resulting in low quantity of nitrite ions. In this assay, 10 mM sodium nitroprusside in phosphate buffered saline was mixed with different concentrations of samples and incubated for 150 min at room temperature. After incubation, Griess reagent (0.5 mL) was added and absorbance was taken at 546 nm by a spectrophotometer. The experiment was repeated in triplicate. Catechin was used as positive control.

#### 2.4.9. Reducing Power Assay

Reducing power was determined following modified protocol reported by [25]. The reaction mixture was prepared by the addition of 100 *μ*L of test samples (12.5, 25, 50, 100, and 200 *μ*g/mL), 100 *μ*L of 200 mM phosphate buffer (pH 6.6), and 100 *μ*L of potassium ferricyanide (10 mg/mL) followed by incubation at 50°C for 30 min. An aliquot of 0.25 mL of 1% trichloroacetic acid was added. From the mixture, 0.25 mL was mixed with 0.25 mL distilled water and 0.4 mL ferric chloride (0.1% w/v). Absorbance was recorded at 700 nm after 30 min of incubation at room temperature. Increased absorbance is indicative of high reducing power. Gallic acid served as positive control.

#### 2.4.10. Total Antioxidant (Phosphomolybdate Assay)

The total antioxidant capacity of the samples was investigated by phosphomolybdate method of Saeed et al. [25]. An aliquot of 0.1 mL of each sample was mixed with 1 mL of reagent (0.6 M H_2_SO_4_, 0.028 M sodium phosphate, and 0.004 M ammonium molybdate) and incubated for 90 min at 95°C in a water bath. Absorbance was recorded at 765 nm after the mixture cooled to room temperature. Ascorbic acid served as positive control.

#### 2.4.11. DNA Protection Assay

The DNA protection assay was evaluated according to the method reported by Tian and Hua [29]. Plasmid DNA (pBR322 Fermentas) 0.5 *μ*g/3 *μ*L was treated with 5 *μ*L of each sample (100, 50, and 25 *μ*g/mL). In the reaction mixture 4 *μ*L of 30% H_2_O_2_ and 3 *μ*L of 2 mM FeSO_4_ were used for Fenton reaction induction. Untreated DNA, treated DNA with 2 mM FeSO_4_, DNA treated with 30% H_2_O_2_, and DNA treated with 2 mM FeSO_4_ and 30% H_2_O_2_ were run simultaneously as a control. The reaction mixture was incubated at 37°C for 60 min. Bromophenol blue (3 *μ*L) as loading dye was added to each reaction mixture after incubation. Samples were run on 1% agarose gel containing ethidium bromide and TBE buffer and visualized with Doc-IT. The experiment was performed in the dark to avoid photoexcitation of samples.

### 2.5. Statistical Analysis

All values are mean of triplicates. One-way ANOVA analysis was carried out by using Statistix 8.1 to assess the difference between various groups. The GraphPad Prism was used to calculate IC_50_ values. Correlation between IC_50_ values of different assays with total flavonoid and total phenolic content was calculated by Pearson's correlation coefficient with GarphPad Prism software version 5. The significance level was *P* < 0.05.

## 3. Results and Discussion

### 3.1. Fractions Yield

*Jurinea dolomiaea* crude methanol extract (JDME) yield was 25% of dry powder, while fractions JDHE, JDCE, JDEE, JDBE, and JDAE yield 55%, 8%, 12%, 10%, and 15%, respectively, of dry crude methanol extract. Extract and fractions yield was recorded differently, depending on the nature of the solvent used. A similar finding was observed in our previous study on* Sida cordata* [22]. In the present study* n*-hexane gave maximum yields and* n*-butanol yields minimum among solvents used.

### 3.2. Total Phenolic and Flavonoids Content

Plant phenolics constitute one of the major classes of compounds which act as natural antioxidants [30]. Therefore it was reasonable to estimate their profile in* J. dolomiaea*. Total phenolic content of* J. dolomiaea* extract and its derived fractions were determined from the standard calibration curve (*R* ^2^ = 0.90) of gallic acid.

Maximum TPC was shown by JDCE (757.0 ± 9.4 mg gallic acid equivalent/g dry sample) followed by JDEE and JDME (630.0 ± 4.5 mg gallic acid equivalent/g dry sample, 620.0 ± 3.8 mg gallic acid equivalent/g dry sample).  Table 1 shows that TPC varied from 10 ± 0.6 mg gallic acid equivalent/g dry sample to 757.0 ± 9.4 mg gallic acid equivalent/g dry sample in different fractions of* J. dolomiaea*. Chloroform proved to be the best solvent in elucidating TPC in maximum quantity from crude methanol extract of* J. dolomiaea*, followed by ethyl acetate solvent. So it is evident from the present data that chloroform and ethyl acetate are the best solvents for fractioning phenolic component from* J. dolomiaea* plant due to their polarity index and the best solubility for the type of phenolic contents in* J. dolomiaea*.* n*-Hexane, which is the lowest polar organic solvent in Table 1, showed lowest phenolic content.

The total flavonoid content of* J. dolomiaea* extract and fractions were estimated from a standard calibration curve of rutin (*R* ^2^ = 0.91).  Table 1 shows that TFC varied from 34.3 ± 1.2 mg rutin equivalent/g dry sample to 807.0 ± 7.2 mg rutin equivalent/g dry sample in different fractions of* J. dolomiaea*. Maximum TFC was shown by JDEE (807.0 ± 7.2 mg rutin equivalent/g dry sample) followed by JDBE (605.0 ± 4.3 mg rutin equivalent/g dry sample) > JDCE (560.0 ± 5.3 mg rutin equivalent/g dry sample) > JDME (420.0 ± 2.5 mg rutin equivalent/g dry sample) > JDAE (210.0 ± 3.9 mg rutin equivalent/g dry sample) > JDHE (34.3 ± 1.2 mg rutin equivalent/g dry sample). All the derived fractions of crude methanol extract showed notably (*P* < 0.05) different quantities of flavonoids showing fine separation by different organic solvents. Ethyl acetate proved to be the best solvent in elucidating TFC in maximum quantity from methanol extract of* J. dolomiaea*, followed by* n*-butanol solvent. So it is exhibited by the present data that ethyl acetate and* n*-butanol are the best solvents for fractioning flavonoid component from* J. dolomiaea* plant due to their high polarity and best solubility for flavonoids. Like TPC fractionation,* n*-hexane also proved to be the lowest flavonoid content bearer in this study.

### 3.3. HPLC Profile of Flavonoids and Phenolics of *J. dolomiaea*

High performance liquid chromatography profile of* J. dolomiaea* crude methanol extract (JDME) and fractions JDEE and JDBE is summarized in Table 2. JDME analysis illustrated two known compounds: caffeic acid and apigenin with maximum quantity of caffeic acid (1.9 *μ*g/mg dry sample). JDEE displayed no reference compound, while JDBE showed 5 reference compounds. The maximum amount was shown by catechin (178.3 *μ*g/mg dry sample) followed by rutin (73.8 *μ*g/mg dry sample). Catechin is an important phenolic compound and has shown many beneficial health associated effects in laboratory as well as in clinics due to their antioxidant activity along with augmenting endogenous antioxidants. Catechin and its metabolites have shown therapeutic potential as neuroprotective, antiapoptotic, and anti-inflammatory in clinical disorders [31]. Caffeic acid is reported for antimetastatic and antitumour activity by suppressing MMP-9 enzyme activity [32]. Rutin is important plant secondary metabolite and reported as hepatoprotective, antioxidant, and anti-inflammatory agent [33]. Presence of high quantity of important secondary metabolites makes* J. dolomiaea* roots a potential bioactive agent.

### 3.4. Antioxidant Capacity

#### 3.4.1. DPPH Radical Scavenging Activity

The IC_50_ values of DPPH radical scavenging activity are given in Table 3. The best IC_50_ was shown by JDEE (41.1 ± 1.0 *μ*g/mL) followed by JDBE (132.9 ± 2.1 *μ*g/mL), while the highest IC_50_ value was shown by JDAE. Overall, the DPPH radical IC_50_ order JDEE < JDBE < JDCE < JDME < JDHE < JDAE was observed. IC_50_ of JDEE was comparable to positive control (ascorbic acid) IC_50_ but significantly different. The DPPH radical scavenging activity of* J. dolomiaea* extract and its various fractions showed good correlation with TFC (*R* ^2^ = 0.74, *P* < 0.05) while nonsignificant with TPC (*R* ^2^ = 0.37, *P* > 0.05).

Though DPPH radical scavenging activity of the fractions is significantly lower (*P* < 0.05) than the standard ascorbic acid, JDEE, JDAE, JDBE, and JDME showed good antioxidant activity and may be attributed to the presence of high quantity of TPC and TFC in these fractions. The antioxidant activity of the phenolic compounds and flavonoids is attributed to the hydroxyl group attached to the aromatic ring which is capable of donating electron and stabilizing free radicals [26].

#### 3.4.2. Hydrogen Peroxide Scavenging Activity

Concentration dependent activity was observed for hydrogen peroxide scavenging. JDEE showed the lowest IC_50_ of 42.2 ± 0.9 *μ*g/mL against hydrogen peroxide, followed by JDBE < JDCE < JDAE < JDME < JDHE (Table 3). IC_50_ of JDEE fraction was significantly lower than ascorbic acid. A highly significant correlation was observed between hydrogen peroxide scavenging activity and TFC (*P* < 0.01, *R* ^2^ = 0.82) while nonsignificant with TPC (*P* > 0.05, *R* ^2^ = 0.21). This shows that overall activity is due to TFC. This difference may be due to the stoichiometry of reactants of both classes resulting in high and low output [34].

#### 3.4.3. Hydroxyl Radical Scavenging Activity

Hydroxyl is short lived, very toxic free radical having affinity to other molecules. It is a very potent oxidizing agent and reacts with a very high rate with most inorganic and organic molecules including lipids, protein, amino acids, sugars deoxyribonucleic acids, leading to cancer, mutagenesis, and cytotoxicity [35, 36]. It reacts with other molecules by hydrogen abstraction, addition, and electron transfer [36].

The hydroxyl radical is generated in chemical reactions in the human body. Superoxide dismutase converts the superoxide oxide into hydrogen peroxide. Hydrogen peroxide is further converted to a highly reactive hydroxyl radical. In the present experiment, the evidence of ^•^OH radical scavenging activity by* J. dolomiaea* extract and its fractions was obtained through the deoxyribose system. Crude methanol extract and derived fractions of* J. dolomiaea* scavenged ^•^OH radicals and prevented 2-deoxyribose breakdown in this assay. A concentration dependent pattern was observed for hydroxyl radical scavenging activity. The lowest IC_50_ values were shown by JDEE and JDBE (47.4 ± 3.3, 77.0 ± 3.5 *μ*g/mL, resp.), while the highest was observed for JDHE (385.0 ± 7.4 *μ*g/mL). IC_50_ of JDEE and JDBE is significantly different from standard ascorbic acid but so near to be comparable. Overall, JDEE < JDBE < JDME < JDCE < JDAE < JDHE hydroxyl radical scavenging IC_50_ order was observed (Table 3). A significant correlation (*R* ^2^ = 0.91, *P* < 0.01) was observed with TFC while nonsignificant (*R* ^2^ = 0.51, *P* > 0.05) with TPC. The strong antioxidant activity of JDEE and JDBE can be utilized as a source of natural antioxidant in oxidative stress for minimizing the detrimental effects of hydroxyl radical in the body.

#### 3.4.4. ABTS Radical Scavenging Activity

Best activity against ABTS radical was shown by JDEE (46.7 ± 0.6 *μ*g/mL) while lowest by JDHE (568.0 ± 4.1 *μ*g/mL) as shown in Table 3. IC_50_ of JDEE was significantly lower than ascorbic acid. Results expressed the concentration dependent activity of all the tested samples. Significant correlation (*P* < 0.01, *R* ^2^ = 0.82) was observed between ABTS radical scavenging activity IC_50_ values with TFC as well as TPC (*P* < 0.05, *R* ^2^ = 0.56) (Table 4).

Arts et al. [37] concluded that evaluation of antioxidant capacity of a compound based not only on chemical structure but also on the type of reaction that is formed. The ABTS scavenging activity of the present study suggests that the component within the extract and various fractions of* J. dolomiaea* are capable of electron/hydrogen donation and should be capable of obstructing oxidative stress [38]. ABTS radical scavenging activity of hydrogen donating antioxidant is observed by Sahreen et al. [24] in their study.

#### 3.4.5. Antilipid Peroxidation Potential

The IC_50_ values of antilipid per oxidation activity of* J. dolomiaea* extract and its various fractions are given in Table 3. Minimum IC_50_ was observed by JDEE and the highest by JDHE with 54.3 ± 1.6 and 2075.0 ± 10.3 *μ*g/mL, respectively. JDEE < JDBE < JDME < JDCE < JDAE < JDHE order of IC_50_ was shown by extract and various fractions. Significant correlation was observed with TFC (*R* ^2^ = 0.64, *P* < 0.05) and nonsignificant with TPC (*R* ^2^ = 0.39, *P* > 0.39). IC_50_ value of JDEE was comparable with standard but significantly different.

#### 3.4.6. *β*-Carotene Scavenging Activity

The antioxidant potential of* J. dolomiaea* extract and different fractions was determined by *β*-carotene bleaching method based on the oxidation of linoleic acid. Solvent polarity based scavenging activity was shown by* J. dolomiaea* extract and its various fractions. Fraction JDEE and JDBE showed the lowest IC_50_ values 82.8 ± 0.6 and 86.5 ± 1.1 *μ*g/mL, respectively, and no significant difference was observed between them. It reveals the presence of some component with different chemistry but showing the same *β*-carotene bleaching activity. The lowest activity was observed by JDAE (267.4 ± 1.3 *μ*g/mL) (Table 3). This study shows that JDEE has notable activity in minimizing the loss of *β*-carotene during the coupled oxidation of linoleic acid and *β*-carotene in the emulsified aqueous system. *β*-Carotene assay showed a significant correlation (*R* ^2^ = 0.74, *P* < 0.05) with TFC and nonsignificant with TPC (*P* > 0.05, *R* ^2^ = 0.35).

#### 3.4.7. Superoxide Radical Scavenging Activity

Compared with other oxygen radicals, superoxide anion has a longer lifetime, can move a long distance, and thus can be dangerous for the affected or associated systems. Therefore, it is very important to study the scavenging of superoxide anion [39].

**Figure 1:**
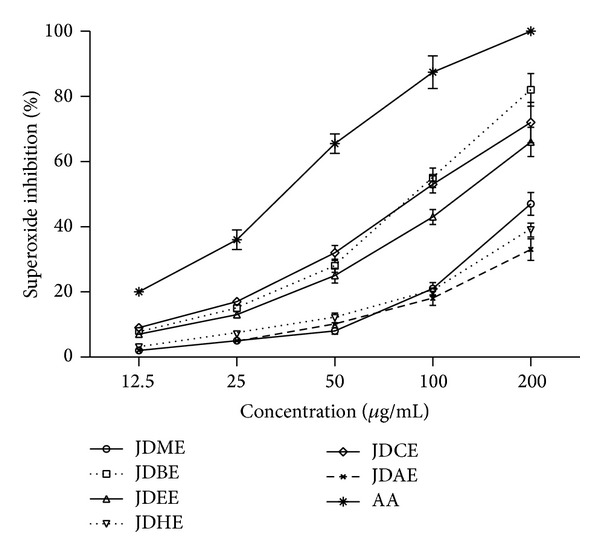
Dose dependent superoxide radical scavenging activity of extract and various fractions of* J. dolomiaea.* Values are expressed as mean ± SD (*N* = 3). JDME:* J. dolomiaea* methanol extract; JDEE:* J. dolomiaea* ethyl acetate fraction; JDBE:* J. dolomiaea n*-butanol fraction; JDAE:* J. dolomiaea* aqueous fraction; JDHE:* J. dolomiaea n*-hexane fraction; JDCE:* J. dolomiaea *chloroform fraction; AA: ascorbic acid used as reference.

Extract as well as all its fractions recorded good superoxide radical scavenging activity. The highest activity was observed from JDCE (91.7 ± 1.3 *μ*g/mL) and the lowest from JDAE (497.8 ± 4.2 *μ*g/mL). Order of JDCE < JDBE < JDEE < JDME < JDHE < JDAE for IC_50_ was estimated from superoxide radical scavenging activity assay (Table 3). TPC as well as TFC showed nonsignificant correlation (*P* > 0.05) with superoxide radical scavenging activity. Our results of superoxide radical scavenging activity are in line with Robak and Gryglewski [40], who say that flavonoids are good antioxidants because they scavenge superoxide radical. Concentration based scavenging effect was observed as shown in Figure 1.

#### 3.4.8. Nitric Oxide Scavenging Assay

In the present study, the lowest IC_50_ value was recorded by JDCE (92.0 ± 1.0 *μ*g/mL) while the highest by JDHE (781.9 ± 4.3 *μ*g/mL). Overall, order of JDCE < JDEE < JDME < JDAE < JDBE < JDHE was observed. IC_50_ of JDCE is higher than positive control but comparable.

It is well known that nitric oxide has an important role in various inflammatory processes. Sustained levels of production of this radical are directly toxic to tissues and contribute to the vascular collapse associated with septic shock, whereas chronic expression of nitric oxide radical is associated with various carcinomas and inflammatory conditions including juvenile diabetes, multiple sclerosis, arthritis, and ulcerative colitis [41]. The present study proved that the extract/fractions of* J. dolomiaea* studied has good nitric oxide scavenging activity and requires further investigation to identify the main agent in pure form.

#### 3.4.9. Reducing Power

**Figure 2:**
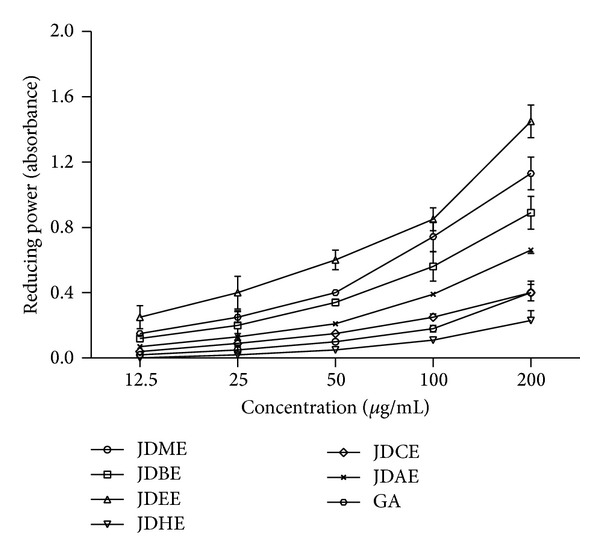
Dose dependent educing power activity of extract and various fractions of* J. dolomiaea.* Values are expressed as mean ± SD (*N* = 3); GA: gallic acid used as reference.

Good reducing power activity was shown by JDEE near to standard gallic acid, followed by JDBE > JDAE > JDMEE > JDCE > JDHE (Figure 2). The phytochemicals present in the JDEE caused reduction of Fe/ferricyanide complex ferrous ion and thus showed reducing power.

Now it is an established phenomenon that reducing power is linked with antioxidant potential and it correlates with phenolic constituents in several vegetables/foods. The reducing power of a compound may serve as an important marker of its possible antioxidant activity. However, the activities of antioxidants have been ascribed to various mechanisms such as prevention of chain initiation, decomposition of peroxides, reducing capacity, and radical scavenging [42].

#### 3.4.10. Total Antioxidant Assay

**Figure 3:**
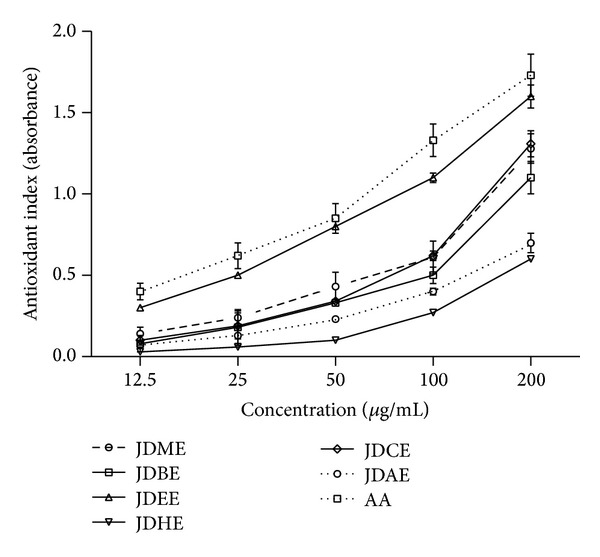
Dose dependent total antioxidant activity of crude methanol extract and various derived fractions of* J. dolomiaea.* Values are expressed as mean ± SD (*N* = 3); AA: ascorbic acid used as reference.

Phosphomolybdenum assay principal follows the chemistry of conversion of Mo (VI) to Mo (V) by compound/extract having antioxidant property and resulting in formation of green phosphate/Mo (V) compound with maximum absorption at 695 nm at acidic pH.* J. dolomiaea* extract as well as its elucidated fractions showed a good antioxidant index as illustrated in Figure 3. Total antioxidant activity order of JDEE (1.60 ± 0.07 *μ*g/mL) > JDCE (1.31 ± 0.08 *μ*g/mL) > JDME (1.28 ± 0.01 *μ*g/mL) > JDBE (1.1 ± 0.1 *μ*g/mL) > JDAE (0.7 ± 0.06 *μ*g/mL) > JDHE (0.6 ± 0.01 *μ*g/mL) was observed at the highest dose of 200 *μ*g/mL. Antioxidant index of JDEE was comparable with ascorbic acid.

Electron and hydrogen transfer from antioxidant compound/extract to Mo (VI) complex occurs in the phosphomolybdenum assay methods. The transfers of electrons or hydrogen depend on different redox potentials in the assays and also depend on the structure of the antioxidant [43]. Phosphomolybdenum method usually detects antioxidants such as carotenoids, ascorbic acid, *α*-tocopherol, and some phenolics, cysteine, and aromatic amines due to hydrogen and electron donating ability [44].

### 3.5. DNA Protection Study

**Figure 4:**
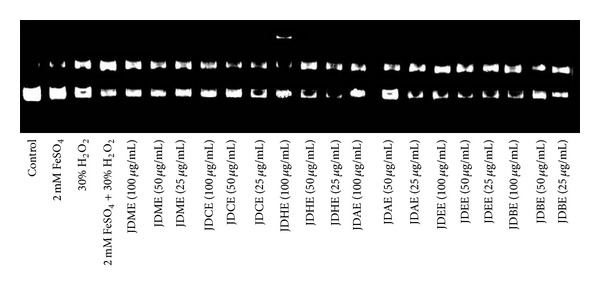
DNA dose dependent (25, 50, and 100 *μ*g/mL) protection assay of* Jurinea dolomiaea* crude methanol extract and its various derived fractions.

In pBR322 DNA gel electrophoretic pattern the band with faster movement represents the native form of super coiled plasmid circular DNA and the band moving slower corresponds to the open circular form (Figure 4). FeSO_4_ and H_2_O_2_ treatment individually showed two bands, but the native form of DNA was more concentrated, indicating a minor damaged form of DNA. However, FeSO_4_ + H_2_O_2_ showed only one band, expressing a damaged form of plasmid DNA. Crude methanol extract and its fraction JDEE showed no protection against the Fenton reaction induced degradation. Fractions JDCE, JDAE, and JDHE showed protection only at high doses. JDBE instead of protection against Fenton reaction showed degrading effect on plasmid DNA at high dose which decreases with drop of concentration.

## 4. Conclusion

Presence of bioactive flavonoids in* J. dolomiaea* roots might be responsible for the DPPH, ^•^OH, ABTS radical scavenging, H_2_O_2_ scavenging, antilipid peroxidation, and anti-*β*-carotene bleaching activity as indicated by the significant correlation of total flavonoid content with IC_50_ values for these diverse systems. Moreover, JDEE illustrated remarkable scavenging activities for DPPH, ABTS, H_2_O_2_, ^•^OH, O_2_^−^, and antilipid peroxidation. These results indicated that* J. dolomiaea* contained potential antioxidant compounds; isolation and characterization of these could provide valuable therapeutic agents for oxidative stress induced human disorders.

## Figures and Tables

**Table 1:** Total phenolic and total flavonoid contents in methanol extract and in different fractions of *J. dolomiaea*.

Extract/fraction	TFC (mg rutin equivalent/g dry sample)	TPC (mg gallic acid equivalent/g dry sample)
JDME	420.0 ± 2.5^d^	620.0 ± 3.8^b^
JDHE	34.3 ± 1.2^f^	10.0 ± 0.6^e^
JDCE	560.0 ± 5.3^c^	757.0 ± 9.4^a^
JDEE	807.0 ± 7.2^a^	630.0 ± 4.5^b^
JDBE	605.0 ± 4.3^b^	247.0 ± 6.2^c^
JDAE	210.0 ± 3.9^e^	113.0 ± 9.8^d^

Values are expressed as mean ± SD (*N* = 3); means with superscripts with different letters in the rows are significantly (*P* < 0.05) different from each other.

**Table 2:** HPLC-DAD profile of *J. dolomiaea* methanol extract and its derived ethyl acetate and *n*-butanol fractions.

Extract/fraction	Flavonoid/phenolics	Signal wavelength	Retention time (min)	Quantity (*μ*g/mg dry sample)
JDME	Caffeic acid	325	9.553	1.9
	Apigenin	325	21.706	0.776

JDBE	Gallic acid	257	4.011	0.77
	Rutin	257	13.104	73.8
	Catechin	279	7.789	178.3
	Caffeic acid	325	9.574	4.59
	Myricetin	368	15.018	3.54

JDEE	No reference compound was observed.			

**Table 3:** IC_50_ values of different antioxidant activities of extract and derived fractions of *J. dolomiaea*.

Activity	IC_50 _(*μ*g/mL)						
	JDME	JDHE	JDCE	JDEE	JDBE	JDAE	Standard
DPPH scavenging activity	194.7 ± 1.3^b^	219.7 ± 2.1^c^	147.2 ± 1.4^d^	41.1 ± 1.0^f^	132.9 ± 2.1^e^	305.2 ± 1.9^a^	25.1 ± 0.8^g^
Hydrogen peroxide scavenging activity	306.7 ± 2.1^b^	409.6 ± 1.7^a^	190.9 ± 1.6^d^	42.2 ± 0.9^g^	118.4 ± 1.1^e^	231.9 ± 1.3^c^	99.0 ± 1.1^f^
Hydroxyl radical scavenging activity	133.0 ± 4.6^d^	385.0 ± 7.4^a^	159.2 ± 4.2^c^	47.4 ± 3.3^f^	77.0 ± 3.5^e^	300.1 ± 5.5^b^	10.2 ± 0.7^g^
ABTS scavenging activity	93.3 ± 2.2^e^	568.0 ± 4.1^a^	191.6 ± 1.5^c^	46.7 ± 0.6^g^	112.5 ± 0.9^d^	324.1 ± 2.1^b^	55.0 ± 1.0^f^
Antilipid per oxidation activity	172.4 ± 3.1^d^	2075.0 ± 10.3^a^	450.1 ± 3.3^c^	54.3 ± 1.6^f^	136.2 ± 1.4^e^	482.5 ± 2.6^b^	31.0 ± 0.6^g^
*β*-Carotene bleaching scavenging activity	114.4 ± 0.8^d^	254.0 ± 1.0^b^	181.7 ± 2.0^c^	82.8 ± 0.6^e^	86.5 ± 1.1^e^	267.4 ± 1.3^a^	35.0 ± 1.8^f^
Superoxide radical scavenging activity	215.1 ± 1.6^c^	257.3 ± 2.1^b^	91.7 ± 1.3^e^	106.7 ± 2.5^d^	83.8 ± 0.7^f^	497.8 ± 4.2^a^	41.0 ± 0.6^g^
NO^−^ radical scavenging activity	237.3 ± 1.3^d^	781.9 ± 4.3^a^	92.0 ± 1.0^f^	131.6 ± 1.4^e^	388.2 ± 4.1^b^	301.3 ± 3.2^c^	40.9 ± 2.3^g^

Values are expressed as mean ± SD (*N* = 3); means with superscripts with different letters in the rows are significantly (*P* < 0.05) different from each other.

**Table 4:** Correlation of IC_50_ values of different antioxidant activities with total phenolic content and total flavonoid content.

Activity	Correlation *R* ^2^	
	TFC	TPC
DPPH scavenging activity	0.74*	0.37
Hydrogen peroxide scavenging activity	0.82**	0.21
Hydroxyl radical scavenging activity	0.91**	0.51
ABTS scavenging activity	0.82**	0.56*
Antilipid per oxidation activity	0.64*	0.39
*β*-Carotene bleaching scavenging activity	0.74*	0.35
Superoxide radical scavenging activity	0.51	0.37
NO^−^ radical scavenging activity	0.58*	0.71*

Values are expressed as mean ± SD (*N* = 3). *,**indicate significance at *P* < 0.05 and *P* < 0.01, respectively.
